# Analysis of the Diffusion Process by pH Indicator in Microfluidic Chips for Liposome Production

**DOI:** 10.3390/mi8070209

**Published:** 2017-07-01

**Authors:** Elisabetta Bottaro, Ali Mosayyebi, Dario Carugo, Claudio Nastruzzi

**Affiliations:** 1 Bioengineering Science Group, Faculty of Engineering and the Environment, University of Southampton, Southampton SO17 1BJ, UK; E.Bottaro@soton.ac.uk (E.B.); A.Mosayyebi@soton.ac.uk (A.M.); 2 Department of Life Science and Biotechnology, University of Ferrara, Via Fossato di Mortara 17, 44121 Ferrara, Italy; 3 Institute for Life Sciences (IfLS), University of Southampton, Southampton SO17 1BJ, UK

**Keywords:** liposomes, microfluidic, mixing, diffusion, pH indicator, microfluidic hydrodynamic focusing

## Abstract

In recent years, the development of nano- and micro-particles has attracted considerable interest from researchers and enterprises, because of the potential utility of such particles as drug delivery vehicles. Amongst the different techniques employed for the production of nanoparticles, microfluidic-based methods have proven to be the most effective for controlling particle size and dispersity, and for achieving high encapsulation efficiency of bioactive compounds. In this study, we specifically focus on the production of liposomes, spherical vesicles formed by a lipid bilayer encapsulating an aqueous core. The formation of liposomes in microfluidic devices is often governed by diffusive mass transfer of chemical species at the liquid interface between a solvent (i.e., alcohol) and a non-solvent (i.e., water). In this work, we developed a new approach for the analysis of mixing processes within microfluidic devices. The method relies on the use of a pH indicator, and we demonstrate its utility by characterizing the transfer of ethanol and water within two different microfluidic architectures. Our approach represents an effective route to experimentally characterize diffusion and advection processes governing the formation of vesicular/micellar systems in microfluidics, and can also be employed to validate the results of numerical modelling.

## 1. Introduction

Nanomedicine is an attractive field involving the production and clinical application of size-controlled nanoparticles, usually employed in therapy as drug delivery systems or in diagnostics as contrast agents [1]. Amongst the different types of nanoparticles, liposomes have attracted considerable interest for their application as drug delivery systems. Liposomes are artificial spherical vesicles generally composed of natural phospholipids, which performance depends on different physico-chemical variables including chemical composition, size and method of production [2]. Different techniques have been developed for producing size-controlled liposomes with reproducible physical properties; the preparation methods can be generally divided into two groups: (i) “bulk” methods, comprising common macroscale or batch techniques; and (ii) microfluidic methods. The first group includes lipid film hydration, solvent (ethanol or ether) injection, and reverse-phase evaporation [3,4].

In recent years, there has been a growing interest in the use of microfluidic-based production of liposome formulations. This approach has proven to be particularly effective, offering several advantages compared to macroscale techniques; these include small amount of reagents required, potential for in-situ analysis at high temporal and spatial resolution, devices’ portability and cost effectiveness [5,6].

In microfluidics, the formation of liposomes is caused by the unfavorable interactions between lipids and water, causing the self-assembly of lipids (a process often defined as nanoprecipitation) to form spherical vesicles [7]. In a typical microfluidic method, phospholipids are dissolved in a polar solvent (e.g., ethanol or isopropanol) and injected in the central channel of a microfluidic hydrodynamic focusing (MHF) device. The solvent is subsequently focused by water streams coming from two lateral channels [8], leading to controlled mixing between chemical species.

Therefore, the formation of liposomes in microfluidic devices is often governed by diffusive mass transfer of compounds at the liquid interface between solvent (i.e., alcohol) and non-solvent (i.e., water). The alcohol, in which the lipids are initially solubilized, diffuses into the water until it decreases down to a critical concentration [9]. The alcohol diffusion thus governs the formation of vesicles, by a mechanism described as “self-assembly”. Specifically, it has been postulated that the reduction of lipids’ solubility associated with water and alcohol diffusion across the fluid streams causes the formation of intermediate structures, in the form of oblate micelles, which finally enclose to form liposomes.

It is well known that microfluidic systems are characterized by steady laminar flow, which typically occurs when the Reynolds number is lower than a critical value of ~2000, due to the stronger contribution of viscous forces compared to inertial forces at the micrometer scale [10]. The laminar flow regime has two main implications: (i) the flow in microchannels is typically characterized by parabolic fluid velocity profile; and (ii) the transfer of chemical species is dominated by diffusion, due to the low fluid velocity magnitude (resulting in low Péclet number) [11].

The diffusion of chemicals (i.e., solvents, solutes and suspended particles) depends on the contact area between the fluids flowing in the microchannels. The diffusion coefficient scales approximately with the inverse of the molecular size (i.e., the hydrodynamic radius) and also depends, to some extent, on the shape of the molecule [12]. Therefore, smaller molecules have higher diffusion coefficient and will move a longer average distance per unit time, compared to larger molecules that have a smaller diffusion coefficient.

On one hand the mixing of chemical species in microfluidic channels is therefore highly controllable (i.e., being governed by diffusion) and reproducible (i.e., due to the laminar flow conditions), but on the other hand, it is associated with low throughput and in some cases full mixing may not be achieved within the limited length typical of microfluidic devices.

Different methods for quantifying mixing in microfluidics have been presented; these are generally based on the acquisition of microscopic images of two or more colored or fluorescently labelled liquids, followed by quantification of mixing efficiency using simple mathematical functions. Examples of dyes employed are food dyes or stains for biological microscopy [13], or fluorescent dyes such as fluorescein [14,15].

Usually, mixing is quantified by processing a set of microscope images to yield a meaningful index, frequently defined as ‘mixing index’ that is representative of the extent of mixing. Different fluids are usually distinguished based on differences in the light intensity and spectral properties received by a charge-coupled device (CCD) camera.

A dye is often used to absorb transmitted light, reflect incoming light, or emit light. The mixing index is computed using intensities of pixels over a cross-section of a grayscale image that delineates a mixing event or region. The simplest index is calculated by taking the standard deviation of the pixel intensities. This method, however, may not be suitable for comparing mixing efficiency across different studies, from the moment that it is sensitive to different lighting conditions that may be difficult to standardize [16].

One approach to measure the concentration of chemical species in microfluidic mixers is based on the use of fluorescent probes, where mixing is assessed from changes in the fluorescence intensity distribution along the device [17]. Three-dimensional characterization of the mixing performance could be performed with these methods, by using confocal microscopes. Alternative techniques based on changes in the fluorescence lifetime of viscosity-sensitive molecular rotors have also been reported [18]. They, however, require expensive equipment, including sensitive detectors, suitable microscope optics, and specific software/hardware, which hinders their adoption from the broader microfluidic and lab-on-a-chip community.

However, methods based on the use of a dye or fluorescent probe typically do not provide a direct quantification of the mixing between a solvent and a non-solvent, but rather a quantification of the transport of a selected dye or probe molecule. The physical and chemical properties of the probe may therefore have a significant impact on the measured mixing performance.

In this study, we describe and critically analyze a new method for studying mixing processes in different microfluidic chip architectures for nanoparticle production (i.e., MHF or Y-junction), which is based on the use of the pH indicator bromoxylenol blue (BB). The method provides a direct quantification of the exchange between solvent and non-solvent, and it relies on the color shift of a pH sensitive molecule, rather than on color or fluorescence intensity changes.

## 2. Experimental and Numerical Methods

### 2.1. Chemicals

Highly pure phosphatidylcholine (PC) 90% from soybean (Phospolipon 90G) was purchased from Lipoid GmbH (Ludwigshafen, Germany). Dimethyldioactdecylammoniumbromide (DDAB), bromoxylenol blue (BB), trichloro(1H,1H,2H,2H)-perfluorooctylsilane, hydrofluoric acid, and ammonium fluoride were purchased from Sigma-Aldrich Co. Ltd (Irvine, UK). Polydimethylsiloxane (PDMS) monomer Sylgard^®^184 and curing agent were purchased from Dow Corning Corporation (Auburn, AL, USA), and SU-8 photoresist from Chestech Ltd (Rugby, UK). All other regents and solvents were supplied by Sigma-Aldrich Co. Ltd (Irvine, UK). The water employed was ultrapure water (Merck Millipore, Billerica, MA, USA).

### 2.2. Microfluidic Devices Design and Fabrication

**Figure 1:**
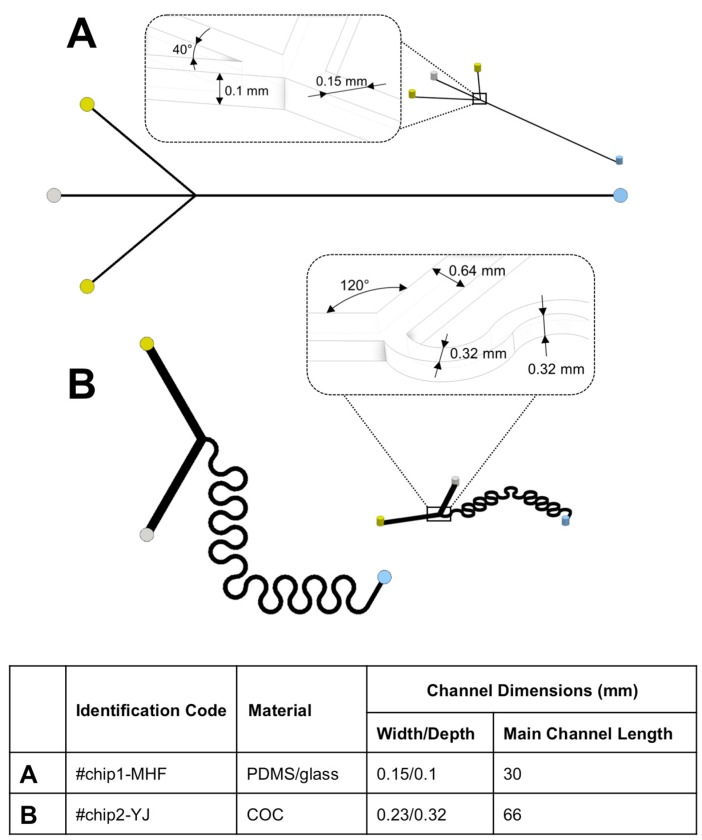
Schematic showing the geometry of the microfluidic chips employed in the present study. (**A**) #chip1-MHF was characterized by a cross flow geometry; while (**B**) #chip2-YJ was characterized by a “Y” shape geometry. The constitutive materials of the chips and the dimensions of the main channel (i.e., located after the junction between the inlet channels) are also reported.

Two different microfluidic architectures were employed in the present study (see Figure 1). #chip1-MHF is characterized by a cross flow geometry, in which the oblique side channels (length: 9.3 mm) intersect the central channel (length: 30 mm) with an angle of 40°. The channels have a rectangular cross section with a width of 0.15 mm and a depth of 0.10 mm. They were produced via soft lithography. Briefly, a SU-8 mold with the designed microchannel architecture was fabricated following a standard procedure [19]. The mold was subsequently covered with a layer of a 10:1 (*w*/*w*) polydimethylsiloxane (PDMS) monomer and curing agent liquid mixture, and heated for 1 hour at 80°. The solidified PDMS sheet, with the microchannel architecture on one surface, was then removed from the mold and permanently bonded to a glass slide after surface treatment with a plasma asher (PVA TePla AG, Wettenberg, Germany).

#chip2-YJ is characterized by a “Y” shape geometry in which the 2 inlets intersect with a 120° angle; the mixing channel (length: 66 mm) has a serpentine geometry. Channels have a squared cross-section with width and depth of 0.32 mm. The device is made of cycloolefin copolymer (COC) and was obtained from Thinxxs Microtechnology (Zweibrücken, Germany).

### 2.3. Liposome Preparation and Characterization

Liposomes were prepared using both #chip1-MHF and #chip2-YJ. The lipid mixture (containing PC 90G at 90 mM, and DDAB at 10 mM) was dissolved in ethanol and injected into the central inlet channel of #chip1-MHF or one inlet of #chip2-YJ; water was instead injected into the two side inlet channels of #chip1-MHF or the second inlet of #chip2-YJ. Teflon^®^ tubes with an internal diameter of 750 µm (Sigma Aldrich, Irvine, UK) were employed to connect the inlets of the devices with syringe pumps (Pump Systems Inc., Farmingdale, PA, USA) for fluids’ delivery.

Liposome formation at different flow regimes was investigated by changing the flow rate ratio between water and ethanol (FRR) in the range 0.5–40, and the total flow rate (TFR) in the range 18.75–75.00 µL/min. The liposome samples were collected from the outlet tube (a 30 mm long Teflon^®^ tube with an internal diameter of 750 µm) in a 1.5 mL microcentrifuge tube. Liposomes were analyzed for size and size distribution by DLS Zetasizer Nano-ZS (Malvern Instruments Ltd, Malvern, UK) with a backscattering detection angle of 173°, a He/Ne laser that emits at 633 nm, and a 4.0 mW power source. The data were used to report the intensity mean diameter (Z-average) and the dispersity of the liposome formulations. The mean particle size was obtained from the results of three independent experiments, carried out at 21 °C in water, without sample dilution (sample volume: 1 mL). Cryo-Transmission Electron Microscopy (cryo-TEM) images of liposomes were also acquired for morphological characterization. For this purpose, a 3 mL aliquot of a liposome sample was applied on plasma-treated (Gatan Solarus Model 950 Advanced Plasma System, pressure = 70 mTorr, H_2_ flow = 6.4 sccm, O_2_ flow = 27.5 sccm, forward RF target = 50 W, exposure time = 30 s) carbon copper grids (Quantifoil R 3.5/1), in the environmental chamber of a fully automated vitrification device for plunge freezing (FEI Vibrot). The relative air humidity was equal to 100% and temperature to 22 °C. The excess solution was removed by blotting with filter paper for 2 s, followed by 1 s draining and plunging of the samples into a 1:1 mixture of liquid ethane and liquid propane, which was cooled to 170 °C. Vitrified samples were cryo-transferred into a Jeol JEM3200FSC cryo-TEM, operating at 194 °C. The temperature of the samples was 187 °C during image acquisition. The microscope was operated in bright field mode, using a 300 kV acceleration voltage; the in-column energy filter was set to 0–20 eV energy-loss range (zero-loss imaging). Micrographs were recorded with a Gatan Ultrascan 4000 CCD camera.

### 2.4. Analysis of Mixing in Microfluidic Chips

The effect of FRR and TFR on the mixing of solvents and solutes in microfluidic channels were studied using the pH indicator BB and NaOH, which were added to the lipid solution and water respectively. BB was added to the ethanol lipid solution until saturation, after adjusting the pH by 0.1 M acetic acid; the concentration of NaOH in water was 0.1 N.

BB is a weak acid, and appears in yellow (below pH 6) or light blue (above pH 7.6) color when it is in the protonated or deprotonated state, respectively. It has a green color in the interval of pH comprised between 6 and 7.6, as an intermediate of the deprotonating mechanism in neutral solution. Therefore, the mixing between ethanol containing BB and water containing NaOH within the microfluidic devices, causes an increase in pH resulting in a change in BB color. 

Different regions of the main channel within the two chips were imaged by an optical microscope (Hund^®^ Wilovert 30, Helmut Hund GmbH, Wetzlar, Germany) equipped with a CCD camera (GXCAM-HICHROMESII, GT-Vision^®^, Haverhill, UK), at 4× magnification.

Images were taken nearby the junction between inlet channels, and at a more distal location along the main channel (in close proximity to the device outlet). The latter position was selected in order to provide a quantification of the overall mixing performance of the devices, at fixed flow dynamic boundary conditions.

Images were processed using ImageJ (NIH, Bethesda, MD, USA), to measure the width of the regions in which BB is either yellow, blue or green.

### 2.5. Numerical Simulation of Fluid and Species Transport

The transport of fluids and chemical species within both microfluidic devices was characterized numerically, using computational fluid dynamic (CFD) simulations. Firstly, the geometry of the microfluidic channels was designed using Inventor Pro 2016 (Autodesk Inc., San Rafael, CA, USA), and then transferred to ICEM CFD 17.0 (Ansys Inc., Concord, MA, USA) for meshing. The fluidic domain was discretized into finite volumes of tetrahedral shape. A mesh dependence study was performed to identify a compromise between solution accuracy and computational cost, leading to an optimal number of mesh volumes of 7′474′063 (#chip1-MHF) and 4′762′651 (#chip2-YJ). These corresponded to a mesh volume edge size of 0.012 mm (#chip1-MHF) and 0.03 mm (#chip2-YJ). Ansys^®^ Fluent 17.0 (Ansys Inc., Concord, MA, USA) was employed to solve for mass and momentum conservation equations (i.e., Navier-Stokes equations at laminar flow regime), and species transfer (i.e., advection-diffusion equations). Boundary conditions were defined so as to replicate the experimental ones; a mass flow boundary condition was imposed at the device inlets, atmospheric pressure was imposed at the outlets, and a no-slip boundary condition was imposed at the channel walls. The experimental values of TFR and FRR were simulated numerically.

Fluids were assumed incompressible and Newtonian, and the ethanol-water diffusion coefficient was set to 1 × 10^−9^ m^2^/s [20]. The effect of solvents’ mixing on fluid density and viscosity was taken into consideration in the simulations. In order to compare the results of numerical simulations with the experimental images, the numerical contours of ethanol mass fraction were transferred to ImageJ for analysis. Stacks of RGB contour images at selected regions of interest (ROI) within the microfluidic devices were converted to 8-bit format, and subsequently thresholded to obtain a binary image. Reference lines were defined in agreement with the experimental image processing protocol, in order to obtain the width of fluid layers of specific relevance for characterizing the mixing process. The physical width of these layers was determined upon appropriate dimensional calibration of the images.

## 3. Results and Discussion

### 3.1. Liposome Preparation

In this study, two different microfluidic chips characterized by different constitutive materials (i.e., PDMS and cycloolefin) and channel architecture were considered (see Figure 1). Notably, one chip was custom built using a design previously developed in our group (#chip1-MHF), while the other was commercially available (#chip2-YJ). They were selected as two relevant model devices employed for the production of nanoparticles and vesicular systems by solvent exchange mechanism [21,22]. Devices’ constitutive materials were compatible with solvents employed in the present study, and the microfluidic channels did not undergo any detectable deformation at the flow rates investigated. Therefore, the mixing performance in these chips can be considered independent from the material properties.

Particularly, #chip1-MHF is characterized by a cross flow geometry, in which the oblique side channels intersect the central channel at an angle of 40°. #chip2-YJ is instead characterized by a “Y” shape geometry in which the two inlets join with a 120° angle; and the main channel has a 66 mm long serpentine geometry.

Both devices were employed for the production of liposomes, composed of PC/DDAB (at a concentration of 9 mM and 1 mM, respectively). Different liposome samples were produced by varying the FRR (from 10 to 50) and maintaining the TFR fixed at 37.5 µL/min. Liposomes were characterized for their size and dispersity by DLS.

**Figure 2:**
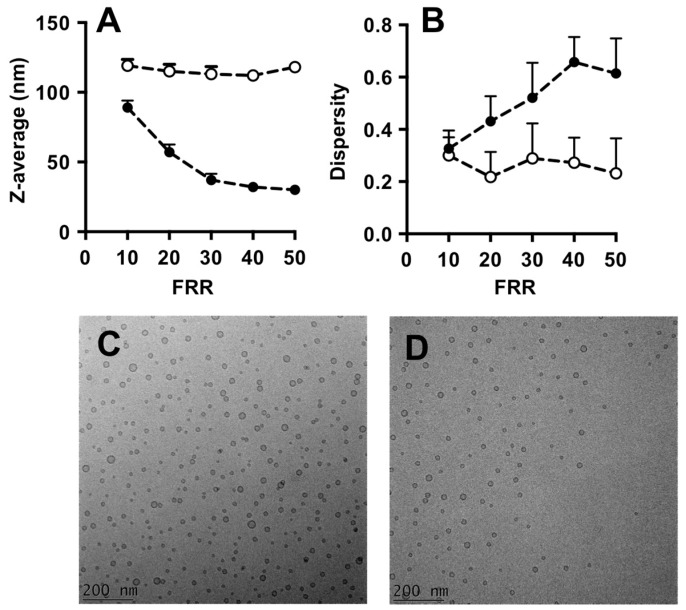
Dimensional characteristics of liposomes produced by microfluidics: Z-average (**A**) and dispersity (**B**). Liposomes were prepared by #chip1-MHF (filled circles) or #chip2-YJ (open circles). Experimental conditions and lipid composition are described in the methods section. Data represent the average of 3 batches, measured in triplicate ± SD; Cryo-TEM images of liposomes produced using #chip1-MHF (**C**) and #chip2-YJ (**D**) are reported, for a FRR of 10 and TFR of 37.5 µL/min.

Data reported in Figure 2 indicate that liposomes produced with #chip1-MHF were generally smaller (ranging between 40 and 110 nm in diameter) than those produced by #chip2-YJ (90–120 nm in diameter).

In addition, an inverse correlation between liposome size and FRR in the microfluidic hydrodynamic focusing device can be appreciated, with an increase in FRR resulting in a decrease in liposome diameter. This is in agreement with previous studies reporting production of liposomes using similar microfluidic architectures [8]. Conversely, the size of liposomes produced with #chip2-YJ did not change significantly with varying the FRR. Previous studies using serpentine shaped microfluidic devices, in which mixing is dominated by advection, have shown that liposome size changed only marginally with increasing FRR [23,24], at values of FRR > 1.

Moreover, increasing the FRR resulted in increased liposome size dispersity for #chip1-MHF, whilst size dispersity was almost independent on FRR for liposomes produced using #chip2-YJ.

### 3.2. Analysis of Mixing in Microfluidic Chips by pH Indicator

**Figure 3:**
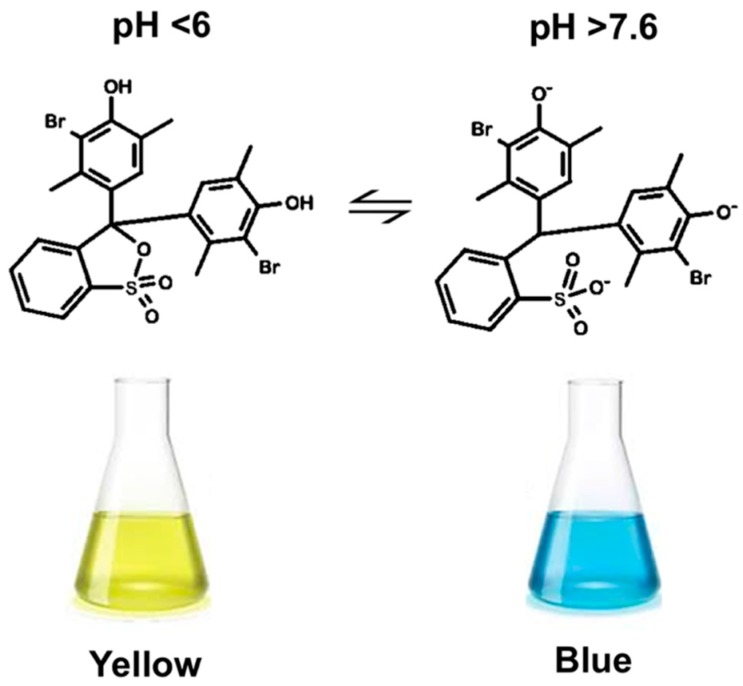
Change in chemical structure and color of bromoxylenol blue (BB) as a function of pH. The color shifts from yellow (at pH < 6) to blue (at pH >7.6).

To characterize the mixing between ethanol and water and its effect on liposome characteristics, a protocol based on the pH indicator bromoxylenol blue was established. BB was selected since it presents a marked, pH-dependent chromatic change that is easily detectable by optical microscopy. At pH < 6 (i.e., the lipid solution in ethanol adjusted with acetic acid) it appears yellow, while at pH > 7.6 (i.e., the NaOH 0.1 N solution in water) it appears blue (Figure 3). For pH values comprised between 6 and 7.6 it has a green color.

Therefore, using BB, the process of mixing in microfluidic devices was analyzed at different flow conditions (i.e., by varying both FRR and TFR). Specifically, FRR was set to 0.5, 2.5, 5.0, 10.0, 20.0 and 40.0, whereas TFR was set to 18.75, 37.50 and 75.00 µL/min.

#### 3.2.1. Microfluidic Hydrodynamic Focusing Device

**Figure 4:**
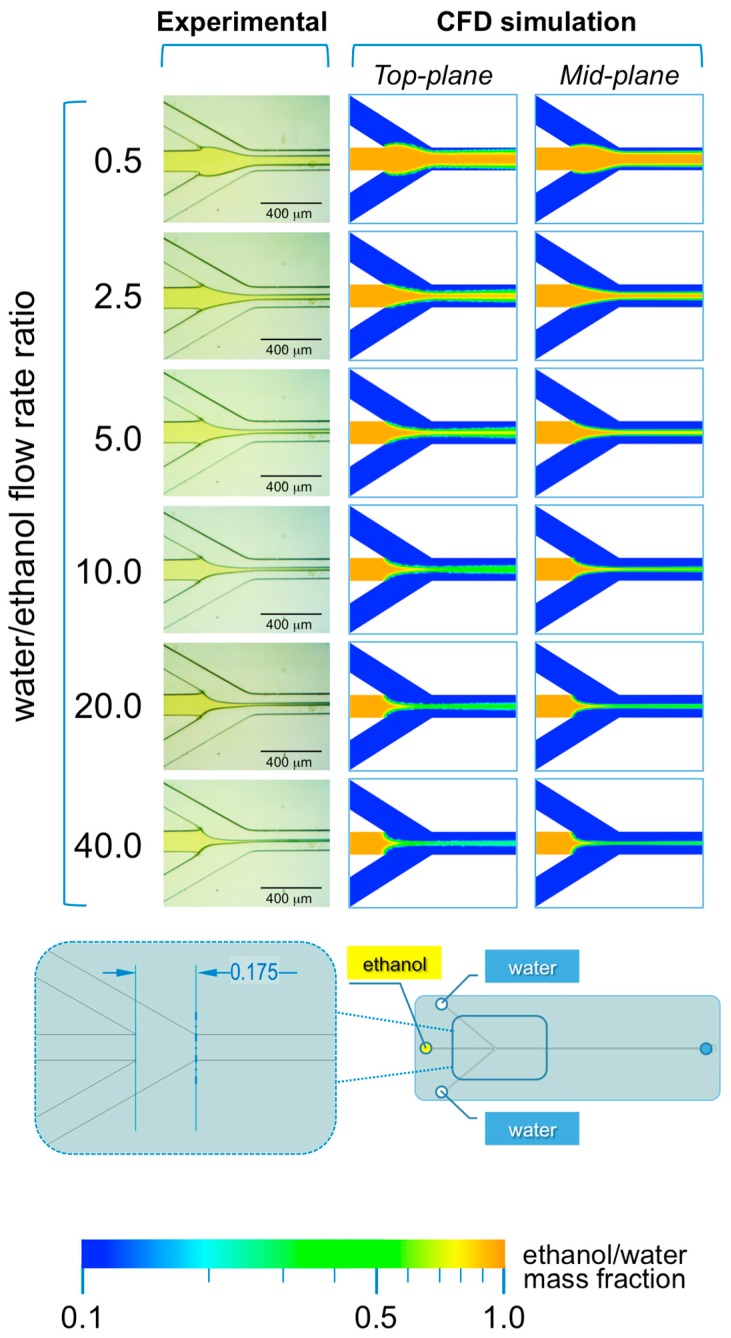
Experimental and computational fluid dynamic (CFD) analysis of the effect of FRR on the shape of the focused stream, in #chip1-MHF. The images illustrate the experimental microscopic observations (**left column**) and the CFD simulations (**mid and right columns**) of the focusing region, at the channels’ intersection; The images were employed to determine the focused stream width at 0.175 mm from the inlet channel, as indicated in the schematic at the bottom; The numerical contours of ethanol mass fraction are reported at both the mid-plane (**mid column**) and top-plane (**right column**) of the device. Experiments and simulations were conducted at TFR of 37.50 µL/min, and at varying FRRs.

**Figure 5:**
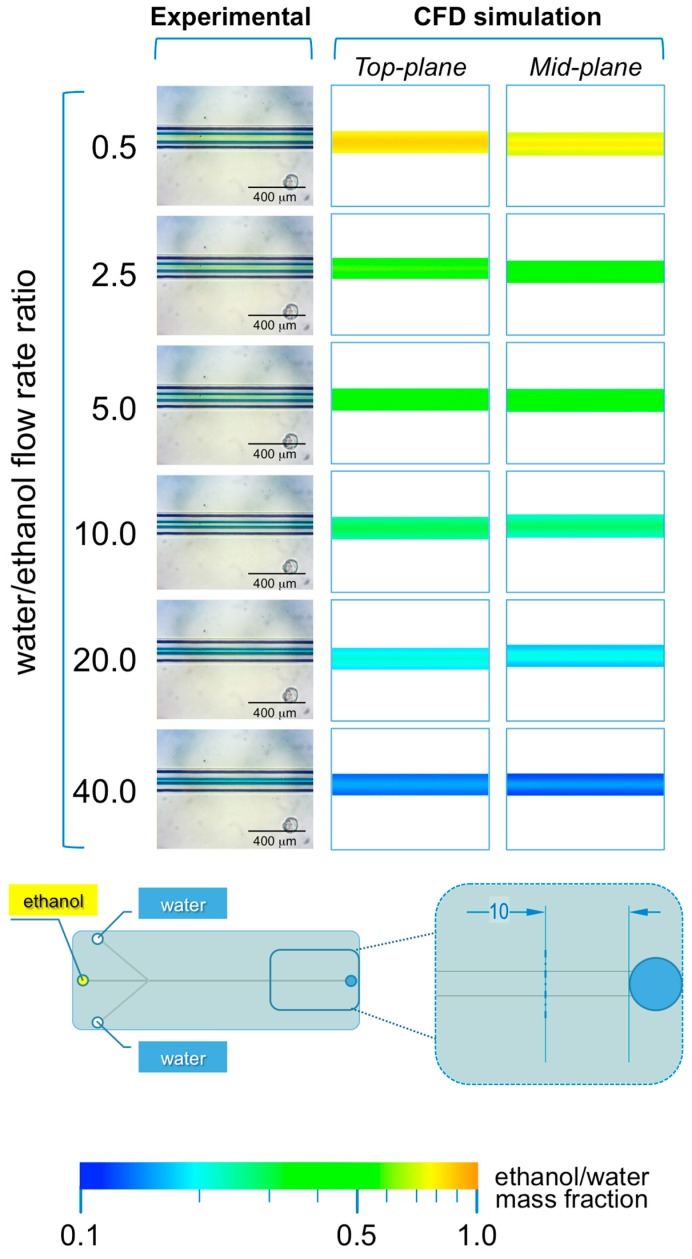
Experimental and computational fluid dynamic (CFD) analysis of the effect of FRR on diffusion and focused stream width, in #chip1-MHF. The images illustrate the experimental microscopic observations (**left column**) and the CFD simulations (**mid and right columns**) of the focusing region, at the channels’ intersection; The images were employed to determine the focused stream width at 10 mm from the outlet, as indicated in the schematic at the bottom; The numerical contours of ethanol mass fraction are reported at both the mid-plane (**mid column**) and top-plane (**right column**) of the device. Experiments and simulations were conducted at TFR of 37.50 µL/min, and at varying FRRs.

The microphotographs taken during the experiments performed with #chip1-MHF at the intermediate TFR value (37.5 µL/min) are reported in Figure 4 and Figure 5, respectively showing the focusing region (i.e., at the inlet channels’ intersection; Figure 4) and the region towards the end of the main channel (i.e., 10 mm from the outlet; Figure 5).

As illustrated in Figure 4, in #chip1-MHF the acidic BB ethanolic solution is hydrodynamically focused by the aqueous NaOH solution into a narrow stream, which width depends on the FRR. Notably, the width of focused stream is inversely correlated to the FRR at all TFRs tested; for instance, at TFR equal to 37.5 µL/min the width progressively decreased from 173 µm (at FRR = 0.5) to 33 µm (at FRR = 40). The numerical simulations are in agreement with the experimental data, with the width of the focused stream decreasing from 143 µm (at FRR = 0.5) to 17 µm (at FRR = 40). These results suggest that the pH indicator approach employed in this study is suitable for characterizing the shape of the focused stream, at the intersection between solvent and non-solvent streams within MHF architectures. Moreover, data suggest that at lower FRRs the larger width of the focused stream may result in higher diffusion length, which is reflected in liposomes having a higher diameter (see Figure 2). Slower mixing however generated dimensionally more uniform liposomes, which is reflected in the lower size dispersity at the lower FRRs (Figure 2). Changes in the local concentration of lipids may have also affected liposome size and size dispersity.

Inversely, higher FRRs produced a narrower focusing of the lipidic ethanolic solution, resulting in a lower diffusion length and therefore faster mixing. Liposomes obtained at these conditions were smaller in diameter, but presented a higher size dispersity (Figure 2). As mentioned earlier, differences in lipid concentration (i.e., due to differences in the ethanol/water ratio) may have also influenced the final liposome size. A compromise between FRR and the concentration/dispersity of liposomes in the end-product should thus be considered when defining the operating conditions of MHF devices.

Cryo-TEM images of liposomes produced with the MHF chip at FRR of 10 and TFR of 37.5 µL/min are reported in Figure 2C.

Notably, the analysis of the mixing in the main channel at 10 mm from the outlet (see Figure 5), suggests that the mixing between the ethanolic solution and water is not complete at all FRRs investigated. This is evident from both experiments and numerical simulations, where excess ethanol in the central stream can be appreciated. This finding suggests that the production of supramolecular assemblies (i.e., liposomes or micelles) by MHF chips with limited channel dimensions, particularly in terms of total length of the main channel, may not be desirable.

Data reported in Figure 5 also indicate that complete mixing of ethanol and water would occur only in the glassware used to collect the samples, therefore diminishing the value of utilizing a highly controlled microfluidic environment. As a matter of fact, the size dispersity of liposomes produced with #chip1-MHF at the conditions reported in the present study was higher when compared with liposomes produced using #chip2-YJ, likely due to inefficient mixing within the microfluidic device. Notably, while the formation of liposomes by solvent exchange is a dynamic process, which kinetics is complex to model or experimentally capture, we can assume that this process reaches an equilibrium once the mixing between solvent and non-solvent is complete. Thus, for the production of liposomes at high throughput, it would be advisable to employ microfluidic chips containing static mixing elements to improve the mixing efficiency between solvent and non-solvent.

**Figure 6:**
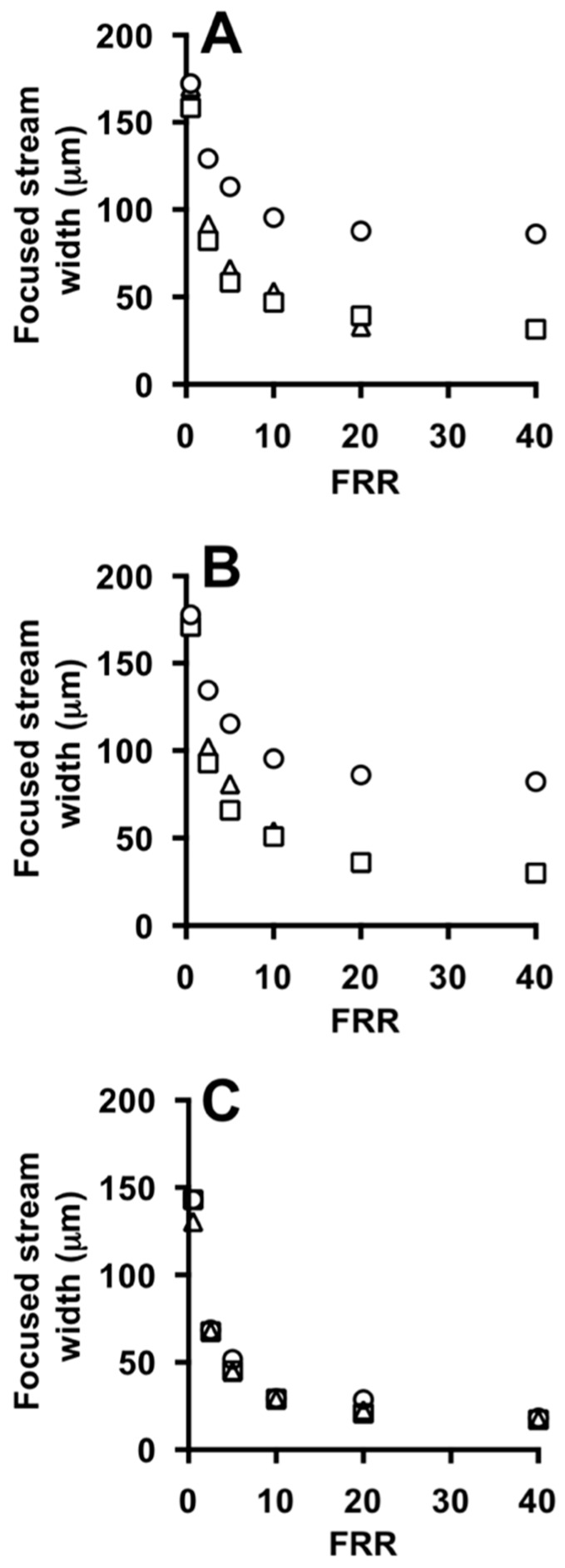
Effect of FFR on the focused stream width at different TFRs, measured from experiments (**A**,**B**) and simulations (**C**) using #chip1-MHF. TFR was set to 18.75 (circles), 37.50 (squares) and 75.00 (triangles) µL/min. Experiments were carried out in the absence (**A**) or in the presence of liposome forming lipids (**B**). Data represent the average of 3 measurements ± SD (the maximum standard deviation is equal to 0.9).

Values of the width of the focused stream are reported in Figure 6A,B, which comprise experiments carried out in the absence and in the presence of phospholipids in the acidic ethanolic solution, respectively. There is no notable effect of having lipids in the ethanolic stream on the shape and size of the focused stream (see Figure 6A,B). It should be noted that the initial total lipid concentration in this study was equal to 100 mM, leading to a final concentration in the range 2–10 mM (depending on the FRR). These values are lower than typical concentrations of commercial formulations, which usually range between 5 mM and 25 mM. This limitation of microfluidic based production methods has been discussed elsewhere more comprehensively. It is envisaged that at these higher lipid concentrations the physical and interfacial properties of fluids may be affected, thus impacting on the size and shape of the focused stream.

Panel C instead compares experimental and numerical data, showing good agreement between the two characterization methods.

#### 3.2.2. Microfluidic ‘Y’-Shape Device

**Figure 7:**
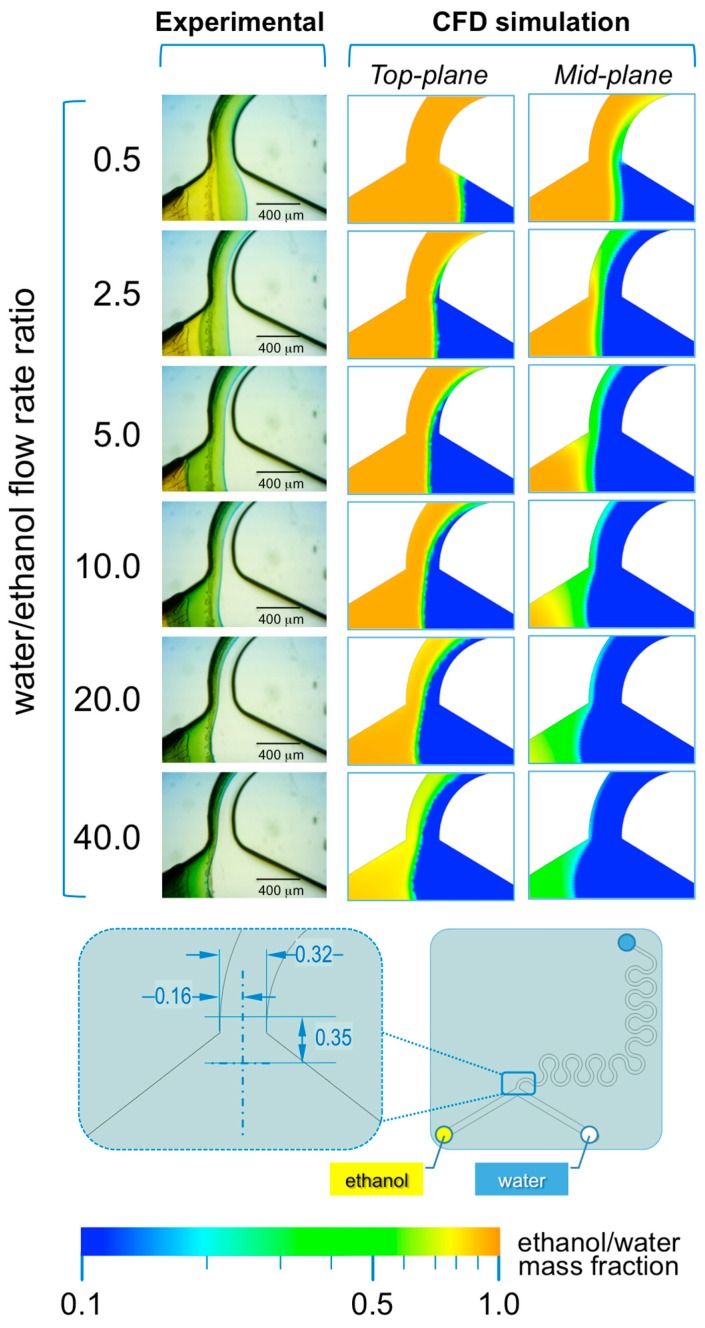
Experimental and computational fluid dynamic (CFD) analysis of the effect of microfluidic parameters on diffusion, diffusion layer width and water/ethanol interface position, in #chip2-YJ. The images illustrate the experimental microscopic observations (**left column**) and the CFD simulations (**mid and right columns**) of the “Y” junction region, at the channels’ intersection; The images were employed to determine the width of the diffusion layer (i.e., the green region) and the water/ethanol interface position, as indicated in the schematic at the bottom; The numerical contours of ethanol mass fraction are reported at both the mid-plane (**mid column**) and top-plane (**right column**) of the device. Experiments and simulations were conducted at TFR of 37.50 µL/min, and at varying FRRs.

In #chip2-YJ the acidic ethanolic solution of the indicator and the aqueous NaOH solution are pumped into the chip from the left and right inlets, respectively (see Figure 7).

In this microfluidic architecture, a single interface is formed between ethanol and water, which position depends on the FRR. As evident from Figure 7, at lower FRRs (in the range 0.5 to 5.0) the interface is shifted towards the right inlet channel, whereas at higher FRRs (>5.0) the interface progressively shifts towards the left inlet channel. This trend is evident in both experimental and simulated conditions, suggesting that simulations are able to capture the interfacial interaction between solvent and non-solvent.

A remarkable difference between #chip1-MHF and #chip2-YJ resides in the dimension of the diffusion layer formed between ethanol and water. In #chip1-MHF, the boundary between solvent and non-solvent appears rather sharp, while in #chip2-YJ a green colored region between ethanol and water is detectable, corresponding to a pH value comprised between 6 and 7.6 (Figure 7). This is reflected in the numerical results, suggesting that mixing between ethanol and water has partially occurred already at the junction between inlets. The width of such diffusion layer appears to be directly related to TFR and inversely related to FRR. Notably, the higher residence time and slower mixing in this specific device resulted in liposomes with larger diameter compared to those obtained with the MHF device, as illustrated in Figure 2.

Furthermore, the numerical results show a significant difference in the solvent concentration between the top and mid planes, likely due to the ethanol moving upwards because of its lower density compared to water. This effect is more evident in #chip2-YJ compared to #chip1-MHF, due to the larger cross-sectional area and therefore the lower mean fluid velocity. However, stratification of fluids due to differences in density did not appear to impact on the mixing efficiency in this specific device. This effect has not been previously investigated in depth, and will form the basis of future studies.

**Figure 8:**
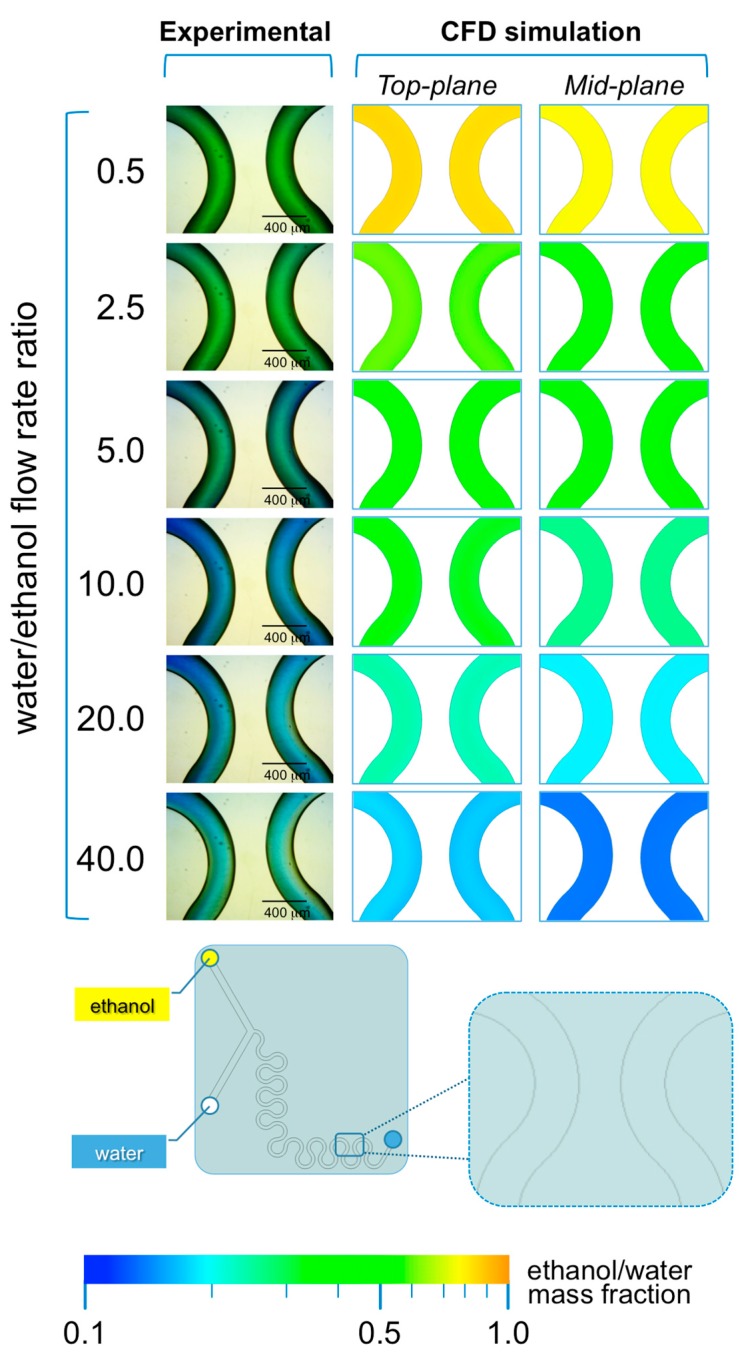
Experimental and computational fluid dynamic (CFD) analysis of the effect of microfluidic parameters on diffusion, diffusion layer width and water/ethanol interface position, in #chip2-YJ. The images illustrate the experimental microscopic observations (**left column**) and the CFD simulations (**mid and right columns**) in a region located at the end of the serpentine geometry; The images were employed to determine the width of the diffusion layer (i.e., the green region) and the water/ethanol interface position, as indicated in the schematic at the bottom; The numerical contours of ethanol mass fraction are reported at both the mid-plane (**mid column**) and top-plane (**right column**) of the device. Experiments and simulations were conducted at TFR of 37.50 µL/min, and at varying FRRs.

As illustrated in Figure 8, the presence of serpentine mixing elements in #chip2-YJ is sufficient to achieve efficient mixing between ethanol and water at all FRRs investigated, without any evident interfacial layer near the outlet of the device (Figure 8). As a result, liposome size dispersity was nearly invariant at the different FRRs. The proportion of ethanol and pH are clearly related to FRR; i.e., at the lower FRRs (0.5 and 2.0) the pH is between 6 and 7.6 as reflected in the green color, and at FRR >10 the pH shifts towards basic values (blue colors). Notably, microfluidic architectures such as #chip2-YJ may provide the benefit of efficient mixing and lower size dispersity at the experimental conditions investigated in the present study.

**Figure 9:**
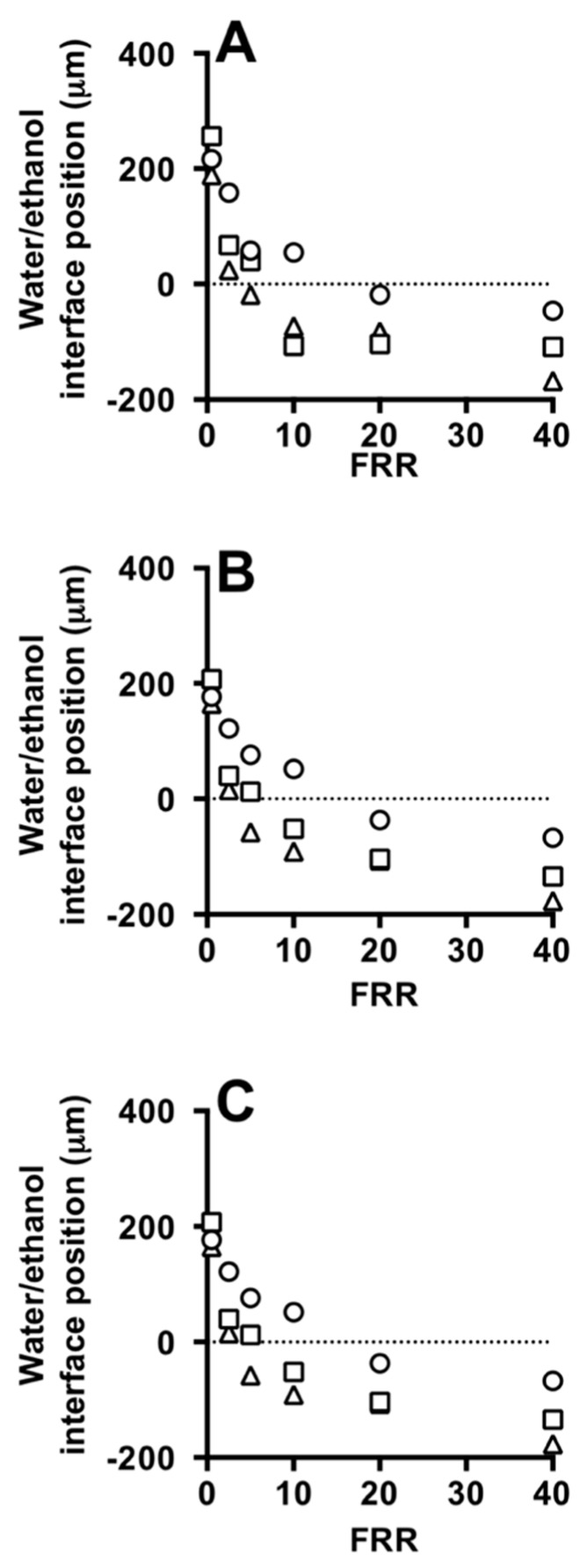
Effect of FFR on the water/ethanol interface position at different TFRs, measured from experiments (**A**,**B**) and simulations (**C**) using #chip2-YJ. TFR was set to 18.75 (circles), 37.50 (squares) and 75.00 (triangles) µL/min. Experiments were carried out in the absence (**A**) and in the presence of liposome forming lipids (**B**). Data represent the average of 3 measurements ± SD (the maximum standard deviation is equal to 0.5).

Quantitative results are provided in Figure 9, showing the influence of FRR on the water/ethanol interface position and diffusion layer width, respectively. In both simulations and experiments, the water/ethanol interface position reduced with increasing FRR. Good agreement between the experimental (Figure 9A) and the computational (Figure 9B) measurements can also be appreciated. Moreover, the presence of lipids in the ethanolic stream does not have a significant effect on the shape and size of this interface (see Figure 9A,B). Cryo-TEM images of liposomes produced with the Y-junction chip at FRR of 10 and TFR of 37.5 µL/min are reported in Figure 2D.

In conclusion, the experimental approach based on the pH indicator BB proved to be effective for studying the influence of FRR and TFR on the mixing performance of microfluidic devices. Notably, the method was validated using numerical simulations, demonstrating its ability to provide a qualitative and quantitative characterization of the mixing between a solvent (ethanol) and a non-solvent (water). The proposed method may provide a useful tool to design and validate appropriate experimental conditions for the use of microfluidic devices in the preparation of supramolecular assemblies, such as liposomes.

## 4. Conclusions

Microfluidic-based production of vesicular systems has proven to be an effective technique, offering several advantages compared to macroscale methods, particularly in terms of control over the physical properties of the end-product. These properties are usually highly dependent on the mixing between a solvent (i.e., ethanol) and a non-solvent (i.e., water). Thus, the design of a microfluidic architecture for vesicular systems’ production requires an in-depth characterization of the mixing within the microfluidic environment. In this study, we report on the development of a novel method based on the use of a pH indicator, and we demonstrated its utility by charactering the transport of solvent and non-solvent within two different microfluidic mixers typically used for the production of vesicular or micellar systems. Numerical simulations were performed to validate the experimental findings. With these methods, we evaluated the effect of the hydrodynamic boundary conditions (specifically the ratio between inlet flow rates, FRR) on the mixing performance of the selected microfluidic architectures, which had distinct geometrical and fluid dynamic characteristics. Our findings suggest that, in MHF devices, particular attention must be paid to the length of the main channel in order to achieve efficient mixing within the microfluidic device. The presence of a serpentine in the main channel was observed to significantly improve the mixing performance, and complete mixing was achieved for the large majority of FRRs and TFRs investigated. The latter device architecture may provide the benefit of efficient mixing at a larger spectrum of FRRs.

Compared to other mixing characterization methods based on changes in color or fluorescence intensity of a dye or probe, our proposed technique relies on the color-shift of a pH sensitive molecule, and may therefore be less sensitive to the lighting conditions employed in the experiment. Furthermore, it provides a route for qualifying and quantifying the solvent exchange process, which is postulated to govern the formation of vesicular systems in microfluidic devices.

The proposed mixing characterization method also presents advantages of cost-effectiveness and easiness of implementation in non-specialised laboratory settings, including those lacking in adequate computational facilities or expertise for performing numerical studies.

## References

[1] Valencia P.M., Farokhzad O.C., Karnik R., Langer R. (2012). Microfluidic technologies for accelerating the clinical translation of nanoparticles. Nat. Nanotechnol..

[2] Soema P.C., Willems G., Jiskoot W., Amorij J., Kersten G.F. (2015). European journal of pharmaceutics and biopharmacutics predicting the influence of liposomal lipid composition on liposome size, zeta potential and liposome-induced dendritic cell maturation using a design of experiments approach. Eur. J. Pharm. Biopharm..

[3] Akbarzadeh A., Rezaei-Sadabady R., Davaran S., Joo S.W., Zarghami N., Hanifehpour Y., Samiei M., Kouhi M., Nejati-Koshki K. (2013). Liposome: Classification, preparation, and applications. Nanoscale Res. Lett..

[4] Walde P. (2004). Preparation of Vesicles (Liposomes).

[5] Sackmann E.K., Fulton A.L., Beebe D.J. (2014). The present and future role of microfluidics in biomedical research. Nature.

[6] Lee C.Y., Chang C.L., Wang Y.N., Fu L.M. (2011). Microfluidic mixing: A review. Int. J. Mol. Sci..

[7] Bilati U., Allémann E., Doelker E. (2005). Development of a nanoprecipitation method intended for the entrapment of hydrophilic drugs into nanoparticles. Eur. J. Pharm. Sci..

[8] Carugo D., Bottaro E., Owen J., Stride E., Nastruzzi C. (2016). Liposome production by microfluidics: Potential and limiting factors. Sci. Rep..

[9] Zook J.M., Vreeland W.N. (2010). Effects of temperature, acyl chain length, and flow-rate ratio on liposome formation and size in a microfluidic hydrodynamic focusing device. Soft Matter.

[10] Brody J.P., Yager P., Goldstein R.E., Austin R.H. (1996). Biotechnology at low Reynolds numbers. Biophys. J..

[11] Sharp K.V., Adrian R.J., Santiago J.G., Molho J.I. (2002). Liquid Flows in Microchannels. MEMS Handbook.

[12] Baldwin J.G.K.L., Dunlop P.J., Gosting L.J., Kegeles G. (1960). Flow equations and frames of reference for isothermal diffusion in liquids.

[13] Werts M.H., Raimbault V., Texier-Picard R., Poizat R., Français O., Griscom L., Navarro J.R. (2012). Quantitative full-colour transmitted light microscopy and dyes for concentration mapping and measurement of diffusion coefficients in microfluidic architectures. Lab Chip.

[14] Robinson T., Valluri P., Manning H.B., Owen D.M., Munro I., Talbot C.B., Dunsby C., Eccleston J.F., Baldwin G.S., Neil M.A. (2008). Three-dimensional molecular mapping in a microfluidic mixing device using fluorescence lifetime imaging. Opt. Lett..

[15] Fang W.F., Hsu M.H., Chen Y.T., Yang J.T. (2011). Characterization of microfluidic mixing and reaction in microchannels via analysis of cross-sectional patterns. Biomicrofluidics.

[16] Verstraeten D., Schrauwen B. (2009). On the Quantification of Dynamics in Reservoir Computing. Artificial Neural Networks—ICANN 2009.

[17] Jahn A., Vreeland W.N., Gaitan M., Locascio L.E. (2004). Controlled vesicle self-assembly in microfluidic channels with hydrodynamic focusing. J. Am. Chem. Soc..

[18] Casadevall X., Srisa-art M., Andrew J., Edel J.B. (2010). Mapping of fluidic mixing in microdroplets with 1 µs time resolution using fluorescence lifetime imaging. Anal. Chem..

[19] Qin D., Xia Y., Whitesides G.M. (2010). Soft lithography for micro- and nanoscale patterning. Nat. Protoc..

[20] Hills E.E., Abraham M.H., Hersey A., Bevan C.D. (2011). Diffusion coefficients in ethanol and in water at 298 K: Linear free energy relationships. Fluid Ph. Equilib..

[21] Ahn Y.-C., Jung W., Chen Z. (2008). Optical sectioning for microfluidics: Secondary flow and mixing in a meandering microchannel. Lab Chip.

[22] Carboni M., Capretto L., Carugo D., Stulz E., Zhang X. (2013). Microfluidics-based continuous flow formation of triangular silver nanoprisms with tuneable surface plasmon resonance. J. Mater. Chem. C.

[23] Kastner E., Kaur R., Lowry D., Moghaddam B., Wilkinson A., Perrie Y. (2014). High-throughput manufacturing of size-tuned liposomes by a new microfluidics method using enhanced statistical tools for characterization. Int. J. Pharm..

[24] Kastner E., Verma V., Lowry D., Perrie Y. (2015). Microfluidic-controlled manufacture of liposomes for the solubilisation of a poorly water soluble drug. Int. J. Pharm..

